# Locked nucleic acid-inhibitor of miR-205 decreases endometrial cancer cells proliferation *in vitro* and *in vivo*

**DOI:** 10.18632/oncotarget.12043

**Published:** 2016-09-15

**Authors:** Anna Torres, Joanna Kozak, Agnieszka Korolczuk, Dominika Rycak, Paulina Wdowiak, Ryszard Maciejewski, Kamil Torres

**Affiliations:** ^1^ Laboratory of Biostructure, Chair and Department of Human Anatomy, Medical University of Lublin, Jaczewskiego 4, 20-090, Lublin, Poland; ^2^ Department of Clinical Pathomorphology, Medical University of Lublin, Jaczewskiego 8, 20-090, Lublin, Poland; ^3^ Chair and Department of Human Anatomy, Medical University of Lublin, Jaczewskiego 4, 20-090, Lublin, Poland

**Keywords:** miR-205-LNA-inhibitor, endometrial cancer, in vivo, mice xenograft, locked nucleic acid

## Abstract

Pathogenesis of endometrial cancer has been connected with alterations of microRNA expression and in particular miR-205 up–regulation was consistently reported in this carcinoma. Presented study aimed to investigate if inhibition of miR-205 expression using LNA-modified-nucleotide would attenuate endometrial cancer cells proliferation *in vitro* and *in vivo*.

In the course of the study we found that the proliferation of endometrial cancer cells (HEC-1-B, RL-95, KLE, Ishikawa) transfected with LNA-miR-205-inhibitor and evaluated using real time cell monitoring as well as standard cell proliferation assay, was significantly decreased. Next, LNA-miR-205-inhibitor was used to assess the *in vivo* effects of miR-205 inhibition of endometrial cancer growth. Cby.Cg-Foxn1<nu>/cmdb mice bearing endometrial cancer xenografts were intraperitoneally injected with nine dosages of 25mg/kg of miR-205-LNA-inhibitor or scramble control or phosphatase buffered saline and were observed for 32 days. We found that systemic administration of miR-205-LNA-inhibitor was technically possible, and exerted inhibitory effect on endometrial cancer xenograft growth *in vivo* with only mild toxic effects in treated animals.

In conclusion our results suggest that systemic delivery of miR-205-LNA-inhibitor is feasible, devoid of significant toxicity, and could be a promising treatment strategy for endometrial cancer. Therefore it warrants further studies in other animal models.

## INTRODUCTION

Endometrial cancer (EC) is the most common malignancy of the female reproductive tract [1, 2]. Although in early clinical stages EC can be successfully treated with surgery alone, a significant number of patients with late-stage disease suffer from high rate of recurrences and death [3].

Pathogenesis of endometrial cancer has been recently connected with alterations of micoRNAs expression and our previous studies detected several microRNAs that were up-regulated in endometrial cancer tissues, with miR-205 being one of the most highly up-regulated [4, 5, 6]. This observation is also concordant with the findings of other research groups, as miR-205 was consistently found increased in endometrial cancer, both in endometrioid and serous types [5-12].

The functional role of miR-205 in endometrial cancer has not been fully revealed. Zhang et al. suggested it could act through inhibiting phosphatase and tensing homologue protein and Su et al. found that miR-205 promoted tumor proliferation and invasion through targeting estrogen-related receptor gamma [13, 14]. Based on our own results and findings presented by other authors we hypothesized that inhibition of miR-205 would decrease the growth of endometrial cancer. Therefore in the present study we aimed to investigate *in vitro* and *in vivo*, if inhibition of miR-205 would limit proliferation of endometrial cancer cells. Locked Nucleic Acid (LNA) oligonucleotides were chosen to elicit inhibition, as they were previously successfully used in studies performed in mice and non-human primates [15, 16]. In order to simulate the mode of treatment administration in humans and evaluate the potential of this therapeutic modality in translational setting, we decided to use systemic delivery of miR-205-LNA-inhibitor in immunodeficient mice.

Although few studies attempted to explore miR-205 roles in EC *in vitro*, to our knowledge this is the first report to investigate *in vivo* effect of systemic delivery of LNA-modified-miR-205 inhibitor in this malignancy.

## RESULTS

### Functional impact of miR-205-LNA-inhibitor within studied cell lines

**Figure 1:**
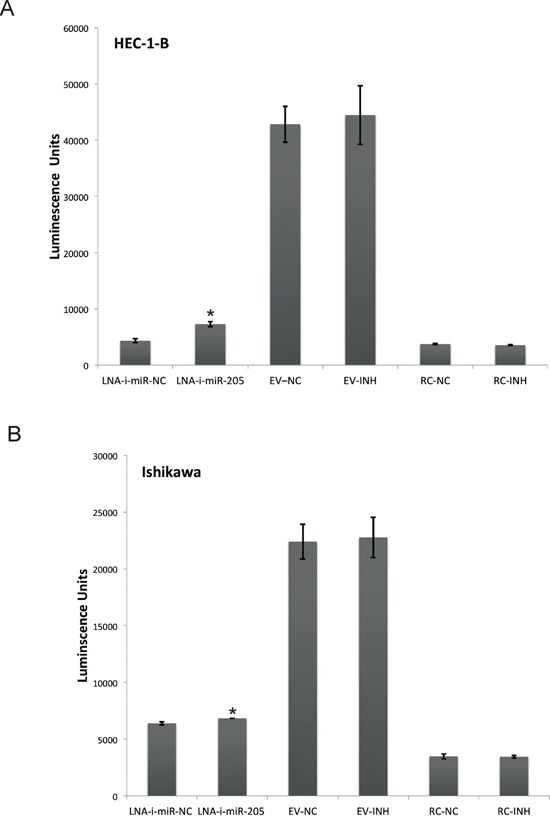
Specificity of LNA-i-miR-205 activity in endometrial cancer cell lines HEC-1-B (p value = 0.021) and Ishikawa (p value = 0.035) based on luciferase activity after co-transfection endometrial cancer lines with pLightSwitch_3′UTR reporter vector and miR-205-LNA-inhibitor; the fluorescence intensity of the empty vector was high HEC-1-B 44 463 AU, SD 520506; Ishikawa 21 767 AU, SD 5206); EV – empty vector, RC – random control. Data are represented as means ± SD from three independent experiments performed in triplicates. * p value < 0.05.

In order to evaluate transfection efficiency of miR-205-LNA-inhibitor and assess its functional impact within studied cell lines (HEC-1-B and Ishikawa) we co-transfected miR-205-LNA-inhibitor or scramble control and pLightSwitch_3′UTR reporter vector containing optimized target sequence complementary to the miR-205-5p (based on miRBase 16) cloned downstream of RenSP luciferase gene. Luciferase activity was significantly increased 24 hours after co-transfection endometrial cancer lines with pLightSwitch_3′UTR reporter vector and miR-205-LNA-inhibitor, proving that the inhibitor was efficient, biologically available and functional within the cells of HEC-1-B and Ishikawa lines (Figure 1). In both cell lines the fluorescence of the empty vector (EV) was significantly higher comparing to miR-205-LNA-inhibitor and scramble control. The fluorescence intensity of the empty vector was 44 463 AU ± SD 5206 in HEC-1-B and 21 767 AU ± SD 1773 in Ishikawa).

### miR-205 inhibits proliferation of EC cells *in vitro*

**Figure 2:**
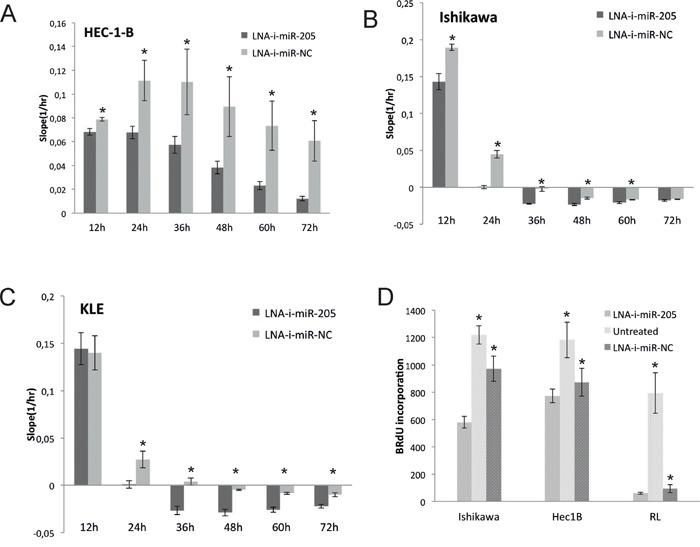
Antiproliferative effect induced by transient transfection of LNA-i-miR-205 in endometrial cancer cell lines compared to scramble control (LNA-i-miR-NC): assessed using *xCELL*igence technology **A.** HEC-1-B: 13% decrease in proliferation was noted 12 hours after transfection, 39% - 24 hours after transfection, 48% - 36 hours after transfection, 57% - 48 hours after transfection, 68% - 60 hours after transfection and 80% - 72 hours after transfection as compared to negative control (p values at 12 to 72h were: 0.004, 0.012, 0.032, 0.026, 0.014, 0.008); **B.** Ishikawa: 24% proliferation inhibition 12 hours after transfection and almost complete proliferation inhibition 24 hours after transfection (p values at 12 to 72h were: 0.0025, 0.0002, 0.0004, 0.0018, 0.01, 0.15); **C.** KLE: 97% decrease in proliferation ability 24h after transfection (p values at 12 to 72h were: 0.78, 0.001, 0.0008, 0.0003, 0.0005, 0.002); **D.** Effects on BrdU uptake: proliferation of Ishikawa cells was reduced by 40% (p values: LNA-i-miR-205 vs. LNA-i-miR-NC – 0.002, LNA-i-miR-205 vs. untreated – 0.0001), HEC-1B cells by 11% (p values: LNA-i-miR-205 vs. LNA-i-miR-NC – 0.04, LNA-i-miR-205 vs. untreated – 0.007) and RL-95 cells by 36% (p values: LNA-i-miR-205 vs. LNA-i-miR-NC – 0.026, LNA-i-miR-205 vs. untreated – 0.001). Data are represented as means ± SD from three independent experiments performed in triplicates. * p value <0.05.

To assess anti-proliferative activity induced by LNA-i-miR-205, we transfected HEC-1-B, Ishikawa and KLE cells and monitored cells proliferation for 72h using *xCELL*igence technology. The transient transfection of LNA-i-miR-205 resulted in a significant inhibition of proliferation in all examined cell lines as compared to LNA-i-miR-NC. Cell proliferation was significantly decreased at all time-points in HEC-1-B line (Figure 2A). Higher-order inhibition of proliferation by LNA-i-miR-205 was observed in Ishikawa cells (Figure 2B). Similar effects were depicted in KLE cells, in which anti-proliferative activity of LNA-i-miR-205 reached significant level 24 hours after transfection (Figure 2C). HEC-1-B cells displayed a 39% decrease in proliferation, while proliferation of Ishikawa and KLE cells was decreased by 99% and 97% 24 hours after transfection as compared to LNA-i-miR-NC, respectively. HEC-1-B cells reached comparable inhibition in proliferation rate 72 hours after transfection.

Proliferation studies based on measurements of BrdU also revealed significant effects of LNA-i-miR-205 in HEC-1B, Ishikawa and RL-95 cell lines (Figure 2D). Similarly to *xCELL*igence experiment higher-order inhibition was observed in Ishikawa comparing to HEC-1-B cells.

### *In vivo* effects of systemic administration of miR-205-LNA-inhibitor

In order to assess *in vivo* effects of intraperitoneal administration of miR-205-LNA-inhibitor we observed treated animals for a total period of 32 days. During that time animals obtained nine dosages of the inhibitor or LNA-i-miR-NC or PBS.

**Figure 3:**
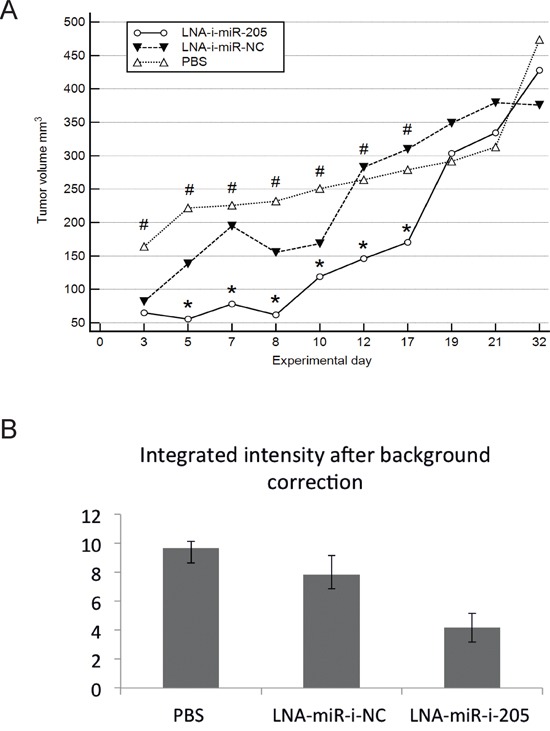
*In vivo* effects of systemic administration of miR-205-LNA-inhibitor **A.** Subsequent measurements of tumor volume showed significant differences between miR-205-LNA-inhibitor, scramble control (LNA-i-miR-NC) and not treated animals (PBS), * p < 0.05 for miR-205-LNA-inhibitor vs. scramble (p values on days 3-17 were: 0.7, 0.036, 0.04, 0.01, 0.045, 0.032, 0.043), # p < 0.05 for miR-205-LNA-inhibitor vs. PBS (p values on days 3-17 were: 0.041, 0.0004, 0.0005, 0.0002, 0.006, 0.027, 0.039); **B.** Scanning with Odyssey Infrared Imaging System revealed markedly reduced fluorescence intensity in the inhibitor group; in addition no visible metastatic sites or extensive infiltration were revealed in either group – graph of representative animals.

Significant inhibition of tumor growth was observed after second injection of miR-205-LNA-inhibitor and was continuously present until the 17^th^ experimental day (after seven dosages of miR-205-LNA-inhibitor). The last two doses did not maintain the effect of the marked inhibition and on the 19^th^ experimental day tumor volumes in PBS and inhibitor groups were of similar size, scramble control was slightly larger but not significantly different. The animals were observed for two weeks following the last dose of the inhibitor to evaluate the natural course of the tumor growth after treatment cessation and to assess possible side or toxic effects of treatment. As it was expected, the tumor growth was accelerated and the final tumor volumes did not differ significantly between control and treatment groups (Figure 3A, Supplementary Figure 1).

### *In vivo* imaging studies

*In vivo* imaging studies were performed on the 15^th^ day of experiment. Scanning tumor images revealed markedly reduced fluorescence intensity in the inhibitor group suggestive of an attenuating effect, consistent with decreased tumor volumes assessed by external measurements. In addition, no visible metastatic sites or extensive infiltration were revealed in either group (Figure 3B).

### miR-205 expression in mice tissues

**Figure 4:**
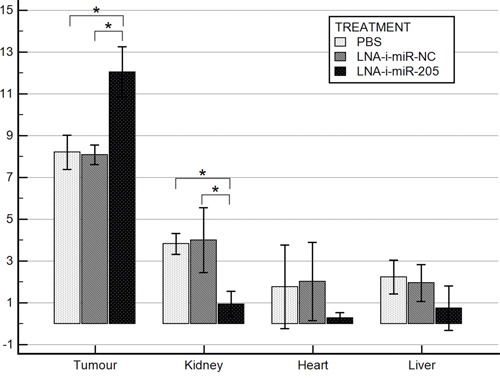
miR-205 expression in mice tissues Bars represent Δ Cq log mean values ± SD; * p value < 0.05 (tumor p < 0.001, post-hoc p < 0.05; kidney p = 0.002, post-hoc p < 0.05; heart p = 0.29; liver p = 0.2).

miR-205 expression was measured in tumors and in vital organs from the studied animals at the end of the experiment. The results were presented in Figure 4. Significantly lower expression of miR-205 was noted in kidneys collected from miR-205-LNA-inhibitor treated mice comparing to animals injected with PBS or scramble control (p = 0.002, post-hoc p < 0.05). miR-205 expression was also lower in heart and liver tissues in treated group, however this difference was not statistically significant.

On the contrary, tumor tissues obtained from miR-205-LNA-inhibitor treated animals demonstrated higher miR-205 expression as compared to PBS and scramble control (*p* < 0.001, post-hoc p < 0.05) (Figure 4).

### Toxicity assessment of systemic administration of miR-205-LNA-inhibitor

During the whole experimental period all animals tolerated procedures well and behaved similarly without any abnormalities, as assessed by activity level, and food and water intake. Body weights were similar in all three groups at the end of the experiment (Table 1). Measurements of vital organs revealed significant increase of spleen weight, and a decrease of heart and uterus weights in the miR-205-LNA-inhibitor group comparing to PBS and scramble control. No differences were found in regards to weight of the liver, kidneys, brain, lungs and ovaries (Table 1). Complete blood counts (CBC) were performed at the end of the experiment and revealed significantly higher levels of hematocrit, red blood cells, white blood cells and platelets as well as slightly lower levels of MCH and MCHC in miR-205-LNA-inhibitor treated animals in comparison to PBS control. However, except for the platelets count, which was elevated in the miR-205-LNA-inhibitor treated animals, the absolute CBC values remained in normal range. Blood parameters of the inhibitor group did not differ significantly from scramble group, except for MCHC, which was slightly lower. There were no significant differences between scramble and PBS groups regarding blood morphology (Table 2).

**Table 1:** Weights of organs collected from experimental animals

Organ weight (g)	LNA-i-miR-205	LNA-i-miR-NC	PBS	*P* value		
	(1)	(2)	(3)	1 vs. 2	1 vs. 3	2 vs. 3
Spleen	0.11	0.08	0.08	0.002	0.001	NS
Liver	1.15	1.13	1.14	NS	NS	
Kidney	0.34	0.3	0.32	NS	NS	
Heart	0.1	0.12	0.13	0.0001	0.0001	
Lungs	0.15	0.14	0.14	NS	NS	
Brain	0.34	0.37	0.38	NS	NS	
Uterus	0.09	0.19	0.16	0.002	0.04	
Ovaries	0.03	0.04	0.04	NS	NS	

NS – not significant.

**Table 2:** Blood cell counts of experimental animals

Parameter	LNA-i-miR-205	LNA-i-miR-NC	PBS	*P* value		
	(1)	(2)	(3)	1 vs. 2	1 vs. 3	2 vs. 3
Hb (g/dL)	13.6	13.2	12.7	NS	NS	NS
RBC (x10^6^/mm^3^)	8.9	8.3	7.85	NS	0.0001	NS
WBC (x10^3^/mm^3^)	3.1	2.5	1.7	NS	0.01	NS
Ht (percent)	45.9	42.7	39.7	NS	0.001	0.006
PLT (x10^9^/L)	954.8	730	733	NS	0.002	NS
MCV (xm^3^)	51.3	51.2	51.7	NS	NS	NS
MCH (pg/cell)	15.2	15.6	16.2	NS	0.02	NS
MCHC (g/dL)	29.7	30.7	31.5	0.01	0.02	NS

NS – not significant.

### Histology studies

**Figure 5:**
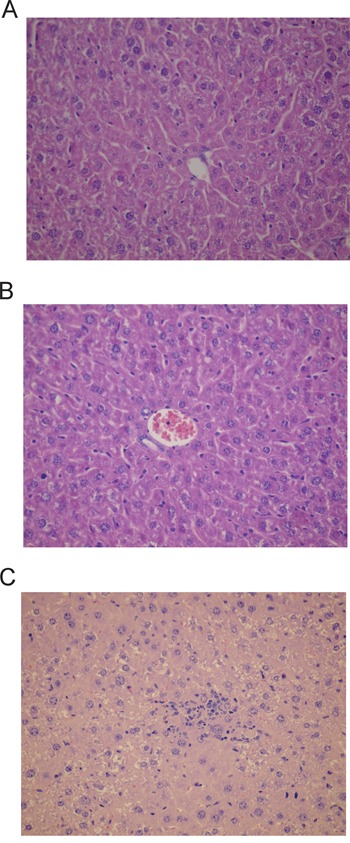
Histological changes in liver encountered after treatment with miR-205-LNA-inhibitor **A.** PBS group-liver architecture within normal limits; **B.** LNA-i-miR-NC group-liver architecture within normal limits; **C.** LNA-i-miR-205 group-small group of hepatocytes necrosis with scattered mononuclear inflammatory cells; slides stained with H+E x 400.

The microscopic examination of heart, spleen, pancreas, lung and brain did not reveal any significant lesions or metastases. The microscopic examination of the liver samples revealed very small, discrete foci of necrosis of small groups of hepatocytes scattered within the lobules that were accompanied by accumulation of mononuclear inflammatory cells. Signs of liver cells regeneration were seen including enlarged nuclei with prominent nucleoli, bi- or trinucleated hepatocytes and scattered mitotic figures. Single apoptotic cells, steatosis of single hepatocytes as well as focal proliferation of Kupffer cells were also observed. The above lesions were present in all animals of miR-205 group. No liver changes were seen in scramble or PBS groups (Figure 5).

## DISCUSSION

Based on our previous studies and results published by other authors we speculated that miR-205 exhibited pro–oncogenic properties in endometrial cancer, and therefore its inhibition with sequence-specific antagonism could be applied as a therapeutic measure in this disease [17]. LNA-modified inhibitors used in the presented study act by forming stable complexes with their target microRNAs, which are then sequestered within the cell [15]. Unique characteristics of LNA-modified oligonucleotides include strong affinity to target sequences, high stability in body fluids as well as resistance to nucleases degradation, and therefore enable their systemic delivery [18]. Moreover, LNA-modified inhibitors were used with success in murine, non-human primate, and human studies, which revealed their long–term safety and efficacy [18, 19, 20].

In the presented study miR-205-LNA-inhibitor (LNA-i-miR-205) effectively inhibited proliferation of endometrial cancer cells *in vitro* [14]. Those results confirmed findings reported recently by Su et al. Proliferation studies reported in our study were performed using two different methodologies. xCELLigence real–time cells proliferation analysis enables monitoring of time-resolved changes in proliferation. We found that inhibition of EC cell proliferation had been increased with time and had been most pronounced 72 hours following transfection in HEC-1-B cells and 48 hours following after transfection in Ishikawa and KLE cells. Subsequent proliferation studies based on measurements of BrdU incorporation confirmed those results. The effect of non-targeting control was quite large in all our experiments in all endometrial cell lines, it was also dose-dependent (data not shown), and therefore we speculate that this might have been due the non-specific effect of the LNA-technology.

Our *in vitro* proliferation studies revealed more significant inhibition of proliferation in Ishikawa cell line, a PTEN-negative line, comparing to HEC-1-B line, which does not exhibit PTEN deficiency. Interestingly, Fong and Meng observed more pronounced response to mTOR inhibitor rapamycin in Ishikawa cells xenografts comparing to HEC-1-A xenografts [21]. In line with those findings Zhang et al. reported, that in Ishikawa cells PTEN was targeted by miR-205 and was decreased by transfection of miR-205 mimic [13]. It is therefore possible that response to miR-205 inhibition could be cell line specific *in vivo* and dependent on PTEN status.

On the basis of our *in vitro* studies and the findings presented by other authors we speculated that systemic delivery of miR-205-LNA-inhibitor could inhibit endometrial cancer growth *in vivo*. The dosing regimen chosen for presented experiment was based on the study by Elmén et al., who reported that LNA inhibitor:miRNA complexes were stable in mouse plasma up to 96 hours [15]. We found that the dose of 25mg/kg administered intraperitoneally three times a week resulted in a substantial decrease of tumor growth, which was significant until the seventeenth experimental day. *In vivo* imaging studies performed on the fifteenth day of the experiment revealed markedly reduced fluorescence intensity in the inhibitor group suggestive of an attenuating effect, consistent with decreased tumor volumes assessed by external measurements.

The last two dosages of the inhibitor did not exert expected attenuation of tumor growth. We speculate that such phenomenon could have been attributed to an inefficient penetration of the inhibitor through the bulk of tumor tissue with intraperitoneal administration. Our hypothesis is supported by the results of Di Martino et al., who found that intravenous administration of LNA-inhibitor in multiple myeloma xenograft bearing mice resulted in a more efficient inhibition of the tumor growth in comparison to intraperitoneal administration [22]. Interestingly, we also found that tumor growth accelerated after treatment cessation in inhibitor treated animals and became similar to non–treated mice at the end of the experiment. Such observation suggested that the inhibitory effect of LNA-i-miR-205 was not long–lasting in tumor tissue and that observed effects needed to be sustained by regular administration of the inhibitor. Another possible explanation would be the development of the rescue mechanisms and resistance to treatment in tumor cells.

miRNA expression studies performed in tissues collected from experimental animals revealed significantly decreased miR-205 levels in kidneys, and markedly (although not significantly) reduced in heart and liver of LNA-i-miR-205 treated mice. This observation suggested that the *in vivo* delivery of the inhibitor was successful and longer–lasting in those organs. Similar effects were reported by Di Martino et al. after systemic delivery of miR-221-LNA-inhibitor in the multiple myeloma bearing mice [23]. Interestingly, we observed significantly higher expression of miR-205 in tumor tissues from treated animals. Such phenomena could be explained by a rebound effect of largely increased miR-205 synthesis in tumor milieu after the period of decreased availability during inhibitor treatment. Increased miR-205 expression at the end of the study was consistent with the observed acceleration of tumor growth, which occurred after cessation of treatment.

Treatment of Cby.Cg-Foxn1<nu>/cmdb mice with LNA-i-miR-205 did not cause any significant adverse or toxic effects as assessed by activity level, food and water intake and histology studies. However, increase of the spleen and decrease of heart and uterus weights were observed, and were accompanied by mild histological changes in liver. Similar toxic effects were observed by Ma et al. after treatment of ovarian cancer bearing mice with miR-10b antagomirs [23]. The authors reported modest changes in spleen and liver sizes, as well as discreet histological changes in liver and decrease in white blood cell and lymphocytes counts [23]. In our study, treatment with miR-205-LNA-inhibitor was linked to elevated hematocrit, red and white blood cells as well as platelets counts. This phenomenon was not seen in PBS and scramble control groups suggesting the possibility of a specific LNA-i-miR-205 mediated activity. In the study performed by Di Martino et al., who evaluated effects of LNA-miR-221 inhibitor in the multiple myeloma bearing mice, no acute or chronic toxicities were found. However, in that study animals were observed for the shorter period of time (two weeks) [23].

There are some limitations to our study, which we plan to address in the future. Firstly, we were not able to evaluate, if alternative root of inhibitor administration i.e. intravenous, would result in a more significant tumor growth inhibition. Secondly, it would be interesting to assess the dose dependent response to LNA-i-miR-205 in terms of tumor growth and systemic toxicity. Lastly, investigation of *in vivo* response in xenografts obtained using other endometrial cancer cells could be of interest, as it was proven in clinical studies, that EC was a heterogeneous entity in regards to response to anti-cancer treatment [24].

In conclusion, the presented study showed that LNA-i-miR-205 was a potent inhibitor of endometrial cancer growth *in vitro*, which was confirmed using four different endometrial carcinoma cell lines. We also demonstrated that systemic administration of miR-205-LNA-inhibitor was feasible and exerted inhibitory effect on endometrial cancer xenograft growth *in vivo* with only mild toxic effects in treated animals. Taken together, these results indicate that systemic delivery of miR-205-LNA-inhibitor could be a promising treatment strategy for endometrial cancer and warrants further studies in other animal models.

## MATERIALS AND METHODS

### Cell lines

Endometrial carcinoma cell lines HEC-1-B, RL-95 and KLE were purchased from ATCC and Ishikawa was purchased from Sigma-Aldrich. Cells were obtained directly from the cell banks, which perform cell line characterizations and passaged in the authors' laboratory for fewer than 6 months after resuscitation. HEC-1-B cell line was maintained in MEM (Gibco) supplemented with 10% fetal bovine serum (FBS), Ishikawa cell line was cultured in MEM (Gibco) supplemented with 5% FBS and KLE and RL-95 were maintained in DMEM (Gibco) supplemented with 10% FBS. All cell lines were maintained with supplementation of 2% penicillin/streptomycin and incubated in humidified chamber at 37°C in 5% CO_2_ atmosphere.

### miR-205-LNA-inhibitor and scramble control

Custom LNA-inhibitor and scramble control (LNA-i-miR-NC) were designed and purchased from Exiqon (Vedbaek, Denmark). Both oligonucleotides had full phosphorothioate (PS) backbones. miR-205-LNA-inhibitor (LNA-i-miR-205) used in our study was custom designed to work effectively in human cells *in vitro* and in mice xenograft of human EC and had a following sequence: CCGGTGGAATGAAGG. LNA-i-miR-NC sequence was as follows ACGTCTATACGCCCA. Oligonucleotides used for the *in vitro* study were FAM labeled. The oligonucleotides were HPLC and Na salt exchanged purity level and were delivered lyophilized. The systemic effects of miR-205-LNA-inhibitor could be reliably evaluated *in vivo*, as sequences of human and murine miR-205 are identical.

### Transfection

LNA-i-miR-205 or LNA-i-miR-NC were incubated with Lipofectamine RNAiMAX at concentration of 12 pmol after dilution in OptiMEM. The mixture was added to cells and seeded into 16-wells (E-16 plate) or 96-wells plate at a density of 2×10^4^ cells per well depending on experiment format. Successful transfection (>50% of all cells) was confirmed by visual fluorescence microscopic analysis.

### RNA isolation from tissues

RNA isolation from tissues stored in RNAlater was performed using mirVANA™ miRNA Isolation Kit (Ambion) according to manufacturer's protocol. Forty milligrams of macro-dissected tissue was used for each isolation. After extraction RNA underwent DNase treatment using Turbo DNAase Kit (Ambion).

### RNA quality control

RNA integrity was checked using Bioanalyzer 2100 (Agilent Technologies Inc.) and Agilent RNA Nano kit. RIN values of RNA ranged between 6 and 8.6. Only samples with RIN≥ 6 were used in downstream applications. Concentration and purity of RNA were measured using spectrophotometry (Biophotometer with Hellma TrayCell, Eppendorf). 260/280 ratio of all RNA samples ranged between 1.8–2.2. All samples were stored at −80°C.

### Reverse transcription and qPCR

#### Reverse transcription

Reverse transcription (RT) was performed using TaqMan^®^ MicroRNA Reverse Transcription Kit and specific primers (Applied Biosystems). RNA extracted from tissues was reverse transcribed in 7.5 μl reactions. Each RT reaction consisted of: 3.5 μL RT Master Mix (0.75 μL 10xRT Buffer, 0.075 μL 100mM dNTPs, 0.5 μL Multiscribe Reverse Transcriptase, 0.095 μL RNase Inhibitor (20U/μL), 2.08 μL Nuclease-free water), 1.5 μL of specific starters and 5 ng RNA in 2.5 μL solution.

The following protocol was used for RT reactions: 16°C for 30 min, followed by 42°C for 30 min and 85°C for 5 min. All RT reactions were carried out in triplicates in Mastercycler ep gradient S (Eppendorf) and stored in −20°C.

#### qPCR

qPCR was performed using single tube TaqMan^®^ MicroRNA Assays and TaqMan^®^ 2 x Universal PCR Master Mix, No AmpErase^®^ UNG (Applied Biosystems) in 10 μl reactions. For miRNA expression analysis in tissues each qPCR reaction consisted of: 0.5 μL 20x TaqMan^®^ MicroaRNA Assay (Applied Biosystems), 4.5 μL RT product (dilution 1:15), 5μL 2x TaqMan^®^ 2x Universal PCR Master Mix (Applied Biosystems).

All qPCR reactions were performed in duplicates in ViiA7 Real–Time PCR System (Applied Biosystems) using protocol suggested by manufacturer. Positive and negative control reactions as well as inter–plate calibrator (IPC) reactions were carried out on each plate. Raw qPCR data were initially normalized with IPCs and adjusted for reaction efficiency. Efficiencies of primer/probe sets were determined by performing standard qPCR with the six-fold dilution of a pool of ten randomly chosen cDNA templates. Efficiencies for all amplicons were calculated using the equation E = 10^(−1/slope)^−1.

For relative quantification of miRNA expression data were normalized using geometric mean of expression of experimentally chosen, stable endogenous controls RNU48, RNU44, U75 and U6, which were previously experimentally validated [25].

### xCELLigence real-time cells proliferation analysis

*xCELLigence* RTCA DP instrument was placed in the humidified incubator at 37°C and 5% CO_2_ atmosphere. Cell proliferation experiments were carried out using E-plates according to manufacturer protocol. Cells were seeded into E-16 plate at a density 2×10^4^ in 100ul per well and experiment was running for 96 hours. Each experiment was performed in triplicate. Data was analyzed using RTCA software and Slope was calculated every 12 hours. The mean of three experiments performed in triplicates at 12, 24, 48 and 72h after transfection were presented in Figure 2.

### Cell proliferation assay

Proliferation of studied cells lines after transfection with LNA-i-miR-205 or LNA-i-miR-NC and Lipofectamine RNAiMAX, was measured using Delfia cell proliferation kit (Perkin-Elmer, Waltham, MA, USA) according to manufacturer protocol. BrdU incorporation was measured by time–resolved fluorescence 48 hours after transfection using VictorX4 multimode plate reader (Perkin-Elmer, Waltham, MA, USA). All experiments were performed in triplicates and repeated tree times.

### Luciferase reporter experiments

In order to additionally evaluate transfection efficiency and assess, if the studied inhibitor had a functional impact within studied cell lines we co-transfected 1×10^6^ EC cells with 50nM miR-205-LNA-inhibitor or scramble control and 30 ng/μl of pLightSwitch_3′UTR reporter vector containing optimized target sequence complementary to the miR-205-5p (based on miRBase 16) cloned downstream of RenSP luciferase gene (Acitve Motif, Carlsbad, CA, US). Twenty four hours following the transfections 100 μg of LightSwitch Assay reagent (Acitve Motif, Carlsbad, CA, US) was added to each well to evoke luciferase reporter signal. The cells were then incubated for 30 minutes in the room temperature and the luminescence was measured using VictorX4 multimode plate reader (Perkin-Elmer, Waltham, MA, USA). The experiments were performed according to manufacture's protocol. The empty pLightSwitch_3′UTR reporter vector (EV) served as a positive transfection control and a negative control for microRNA signaling. We also used pLightSwitch_Random 3′UTR Control 1 (RC) as an additional negative control for microRNA signaling. All experiments were performed in triplicates and repeated tree times.

### Animals and *in vivo* study design

*In vivo* study was conducted in 15 female Cby.Cg-Foxn1<nu>/cmdb mice aged 6 to 8 weeks with the body mass between 16.3 and 20.2 grams. The animals were purchased from Centre of Experimental Medicine, Medical University of Białystok, Poland. The animals were housed in the sterile conditions and were monitored every other day for weight, physical activity and signs of distress. Ethical Committee of Medical University of Białystok approved study design and experimental procedures (# 7/2012).

The xenografts of EC were induced by subcutaneous injection of 50 μl suspension of HEC-1-B cells in PBS (1 x 10^7^ cells) into interscapular area. Mice were randomly divided into three groups (five animals in each group) and were treated with miR-205-LNA-inhibitor, scramble control or PBS, respectively. miR-205-LNA-inhibitor and scramble control were administered intraperitoneally in the dose of 25 mg/kg three times per week. Injections were commenced on the day following the HEC-1-B cells injection. The experiment was conducted for 32 days. After the last injection, which took place on the 20^th^ day of the experiment the mice were left for observation and they were sacrificed by anesthesia performed with 3% isofluran. 0.5 mL of blood was retrieved from the heart of each animal and was preserved in EDTA for blood count. Tissues were collected and half of each organ was preserved for RNA isolation in RNA later and −80°C, and the other half for histology studies in buffered 10% formalin, and processed to paraffin blocks. Four-micrometer slides were cut on the microtome and stained with hematoxylin and eosin.

### *In vivo* imaging

Near IR fluorescence imaging of live animals was performed on the 15^th^ experimental day using Odyssey Infrared Imaging System. Twenty-four hours before imaging the animals were injected with the EGF-IRDye-800CW (epidermal growth factor antibody), which was delivered via tail vein in the dosage of 1nmol/L per mice. The affinity of the antibody to HEC-1-B cells was assessed in the *in vitro* experiment prior to *in vivo* imaging.

### Statistical analysis

qPCR data analysis was performed using GenEx 5.3.4. (MultiD). After normalization, data were log transformed before statistical analysis. Results of functional experiments are presented as means with standard deviation (SD) of three independent experiments performed in triplicates. Comparisons between two independent groups were performed using Student's t test. For comparisons between dependent groups the paired t-test was utilized. For multiple comparisons of independent groups ANOVA test with Tuckey-Kramer post-hoc test was applied. Statistical significance was determined by *P*-value of less that 0.05. Statistical analyses were performed using MedCalc Statistical Software version 14.12.0 (MedCalc Software bvba, Ostend, Belgium; http://www.medcalc.org; 2014).

## SUPPLEMENTARY MATERIALS FIGURES



## References

[1] Siegel RL, Miller KD, Jemal A (2015). Cancer statistics, 2015. CA Cancer J Clin.

[2] Torre LA, Bray F, Siegel RL, Ferlay J, Lortet-Tieulent J, Jemal A (2015). Global cancer statistics, 2012. CA Cancer J Clin.

[3] Bradford LS, Rauh-Hain JA, Schorge J, Birrer MJ, Dizon DS (2015). Advances in the management of recurrent endometrial cancer. Am J Clin Oncol.

[4] Logan M, Hawkins SM (2015). Role of microRNAs in cancers of the female reproductive tract: insights from recent clinical and experimental discovery studies. Clin Sci (Lond).

[5] Torres A, Torres K, Pesci A, Ceccaroni M, Paszkowski T, Cassandrini P, Zamboni G, Maciejewski R (2013). Diagnostic and prognostic significance of miRNA signatures in tissues and plasma of endometrioid endometrial carcinoma patients. Int J Cancer.

[6] Torres A, Torres K, Pesci A, Ceccaroni M, Paszkowski T, Cassandrini P, Zamboni G, Maciejewski R (2012). Deregulation of miR-100, miR-99a and miR-199b in tissues andplasma coexists with increased expression of mTOR kinase in endometrioid endometrial carcinoma. BMC Cancer.

[7] Ratner ES, Tuck D, Richter C, Nallur S, Patel RM, Schultz V, Hui P, Schwartz PE, Rutherford TJ, Weidhaas JB (2010). MicroRNA signatures differentiate uterine cancer tumor subtypes. Gynecol Oncol.

[8] Snowdon J, Zhang X, Childs T, Tron VA, Feilotter H (2011). The microRNA-200 family is upregulated in endometrial carcinoma. PLoS One.

[9] Chung TK, Cheung TH, Huen NY, Wong KW, Lo KW, Yim SF, Siu NS, Wong YM, Tsang PT, Pang MW, Yu MY, To KF, Mok SC (2009). Dysregulated microRNAs and their predicted targets associated with endometrioid endometrial adenocarcinoma in Hong Kong women. Int J Cancer.

[10] Karaayvaz M, Zhang C, Liang S, Shroyer KR, Ju J (2012). Prognostic significance of miR-205 in endometrial cancer. PLoS One.

[11] Qin AY, Zhang XW, Liu L, Yu JP, Li H, Wang SZ, Ren XB, Cao S (2013). MiR-205 in cancer: an angel or a devil?. Eur J Cell Biol.

[12] Hiroki E, Akahira J, Suzuki F, Nagase S, Ito K, Suzuki T, Sasano H, Yaegashi N (2010). Changes in microRNA expression levels correlate with clinicopathological features and prognoses in endometrial serous adenocarcinomas. Cancer Sci.

[13] Zhang G, Hou X, Li Y, Zhao M (2014). MiR-205 inhibits cell apoptosis by targeting phosphatase and tensin homolog deleted on chromosome ten in endometrial cancer Ishikawa cells. BMC Cancer.

[14] Su N, Qiu H, Chen Y, Yang T, Yan Q, Wan X (2013). miR-205 promotes tumor proliferation and invasion through targeting ESRRG in endometrial carcinoma. Oncol Rep.

[15] Elmén J, Lindow M, Silahtaroglu A, Bak M, Christensen M, Lind-Thomsen A, Hedtjärn M, Hansen JB, Hansen HF, Straarup EM, McCullagh K, Kearney P, Kauppinen S (2008). Antagonism of microRNA-122 in mice by systemically administered LNA-antimiR leads to up-regulation of a large set of predicted target mRNAs in the liver. Nucleic Acids Res.

[16] Lindholm MW, Elmén J, Fisker N, Hansen HF, Persson R, Møller MR, Rosenbohm C, Ørum H, Straarup EM, Koch T (2012). PCSK9 LNA antisense oligonucleotides induce sustained reduction of LDL cholesterol in nonhuman primates. Mol Ther.

[17] Fabani MM, Gait MJ (2008). miR-122 targeting with LNA/2′-O-methyl oligonucleotide mixmers, peptide nucleic acids (PNA), and PNA-peptide conjugates. RNA.

[18] Elmén J, Lindow M, Schütz S, Lawrence M, Petri A, Obad S, Lindholm M, Hedtjärn M, Hansen HF, Berger U, Gullans S, Kearney P, Sarnow P (2008). LNA-mediated microRNA silencing in non-human primates. Nature.

[19] van der Ree MH, van der Meer AJ, de Bruijne J, Maan R, van Vliet A, Welzel TM, Zeuzem S, Lawitz EJ, Rodriguez-Torres M, Kupcova V, Wiercinska-Drapalo A, Hodges MR, Janssen HL (2014). Long-term safety and efficacy of microRNA-targeted therapy in chronic hepatitis C patients. Antiviral Res.

[20] Janssen HL, Reesink HW, Lawitz EJ, Zeuzem S, Rodriguez-Torres M, Patel K, van der Meer AJ, Patick AK, Chen A, Zhou Y, Persson R, King BD, Kauppinen S (2013). Treatment of HCV infection by targeting microRNA. N Engl J Med.

[21] Fong P, Meng LR (2014). Effect of mTOR inhibitors in nude mice with endometrial carcinoma and variable PTEN expression status. Med Sci Monit Basic Res.

[22] Di Martino MT, Gullà A, Gallo Cantafio ME, Altomare E, Amodio N, Leone E, Morelli E, Lio SG, Caracciolo D, Rossi M, Frandsen NM, Tagliaferri P (2014). In vitro and in vivo activity of a novel locked nucleic acid (LNA)-inhibitor-miR-221 against multiple myeloma cells. PLoS One.

[23] Ma L, Reinhardt F, Pan E, Soutschek J, Bhat B, Marcusson EG, Teruya-Feldstein J, Bell GW, Weinberg RA (2010). Therapeutic silencing of miR-10b inhibits metastasis in a mouse mammary tumor model. Nat Biotechnol.

[24] Bregar A, Robison K, Dizon DS (2014). Update on the chemotherapeutic management of endometrial cancer. Clin Adv Hematol Oncol.

[25] Torres A, Torres K, Wdowiak P, Paszkowski T, Maciejewski R (2013). Selection and validation of endogenous controls for microRNA expression studies in endometrioid endometrial cancer tissues. Gynecol Oncol.

