# Supplementary material for: Locked nucleic acid-inhibitor of miR-205 decreases endometrial cancer cells proliferation in vitro and in vivo
Source: Oncotarget. 2016 Sep 15;7(45):73651–63. doi: 10.18632/oncotarget.12043 (PMC5342005; doi:10.18632/oncotarget.12043)
Supplement: Supplementary file 1 [file oncotarget-07-73651-s001.pdf]

Locked nucleic acid-inhibitor of miR-205 decreases endometrial cancer cells proliferation *in vitro* and *in vivo*

SUPPLEMENTARY FIGURE

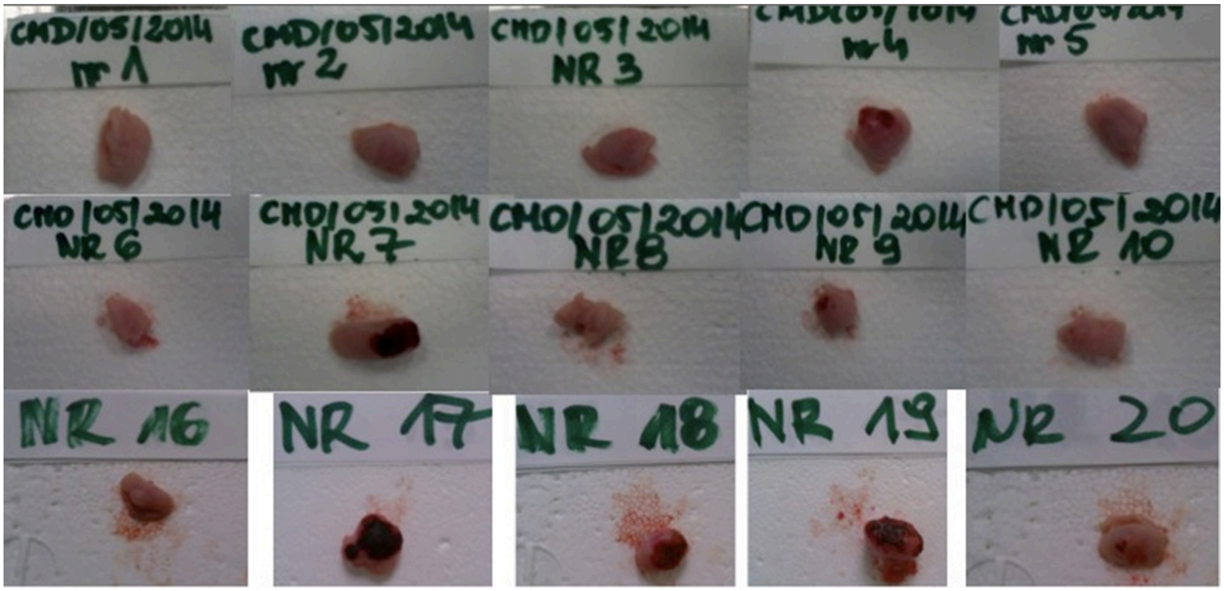

Supplementary Figure S1: Tumors retrieved from experimental animals. Nr. 1 – 5: PBS treated mice; Nr. 6 – 10: LNA-i-miR-NC treated mice; Nr. 16 – 20: LNA-i-miR-205 treated mice.
